# Supplementary material for: GB83, an Agonist of PAR2 with a Unique Mechanism of Action Distinct from Trypsin and PAR2-AP
Source: Int J Mol Sci. 2022 Sep 13;23(18):10631. doi: 10.3390/ijms231810631 (PMC9506296; doi:10.3390/ijms231810631)
Supplement: Supplementary file 1 [file ijms-23-10631-s001.zip › ijms-1902488-supplementary.pdf]

Supporting Information

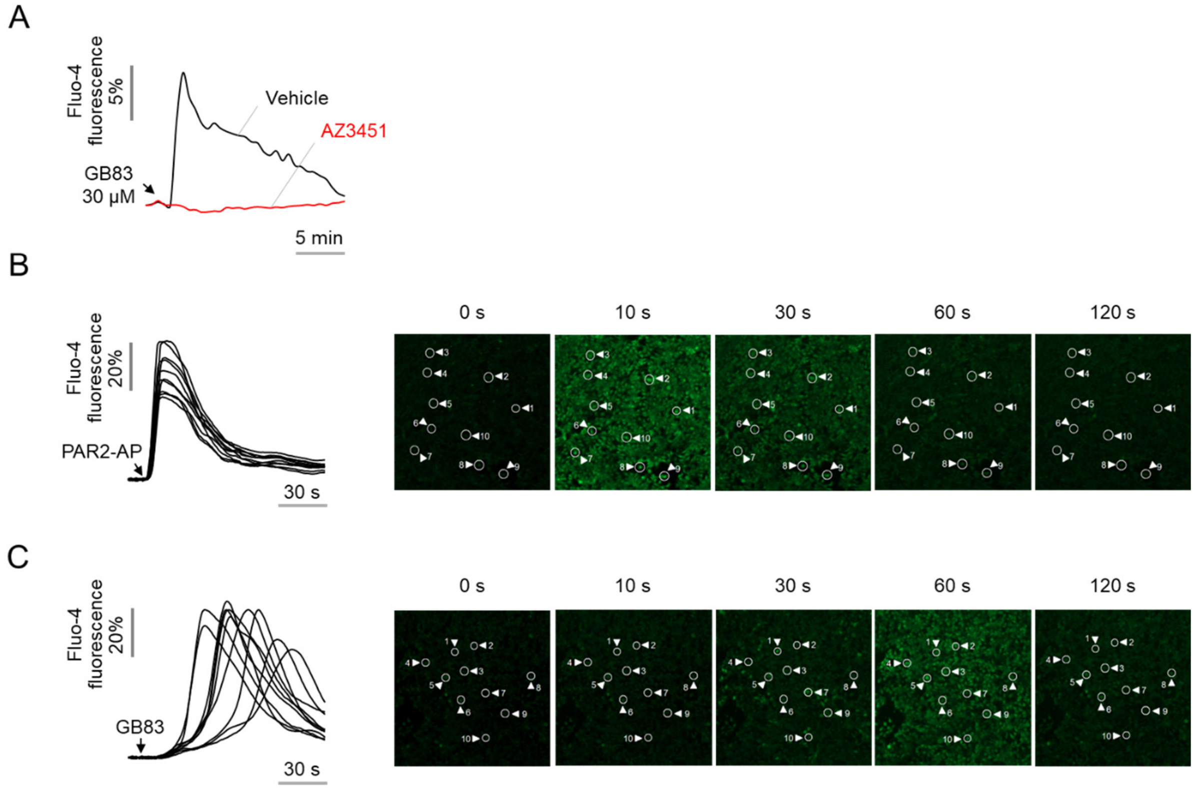

Figure S1. GB83 induced prolonged and asynchronous calcium signaling in HT-29 cells. (A) HT-29 cells were treated with GB83 (30 μM) and intracellular calcium level was monitored using Fluo-4 NW calcium assay kit. AZ3451 (1 μM) was pretreated for 15 min prior to application of GB83. (B-C) Intracellular calcium increase of individual cells in response to PAR2 activation by PAR2-AP and GB83 were monitored using Fluo-4 NW calcium assay kit. Ten single cells were randomly selected and the change in calcium concentration in each cell was observed. Each curve represents change in intracellular calcium concentration in the selected cells.

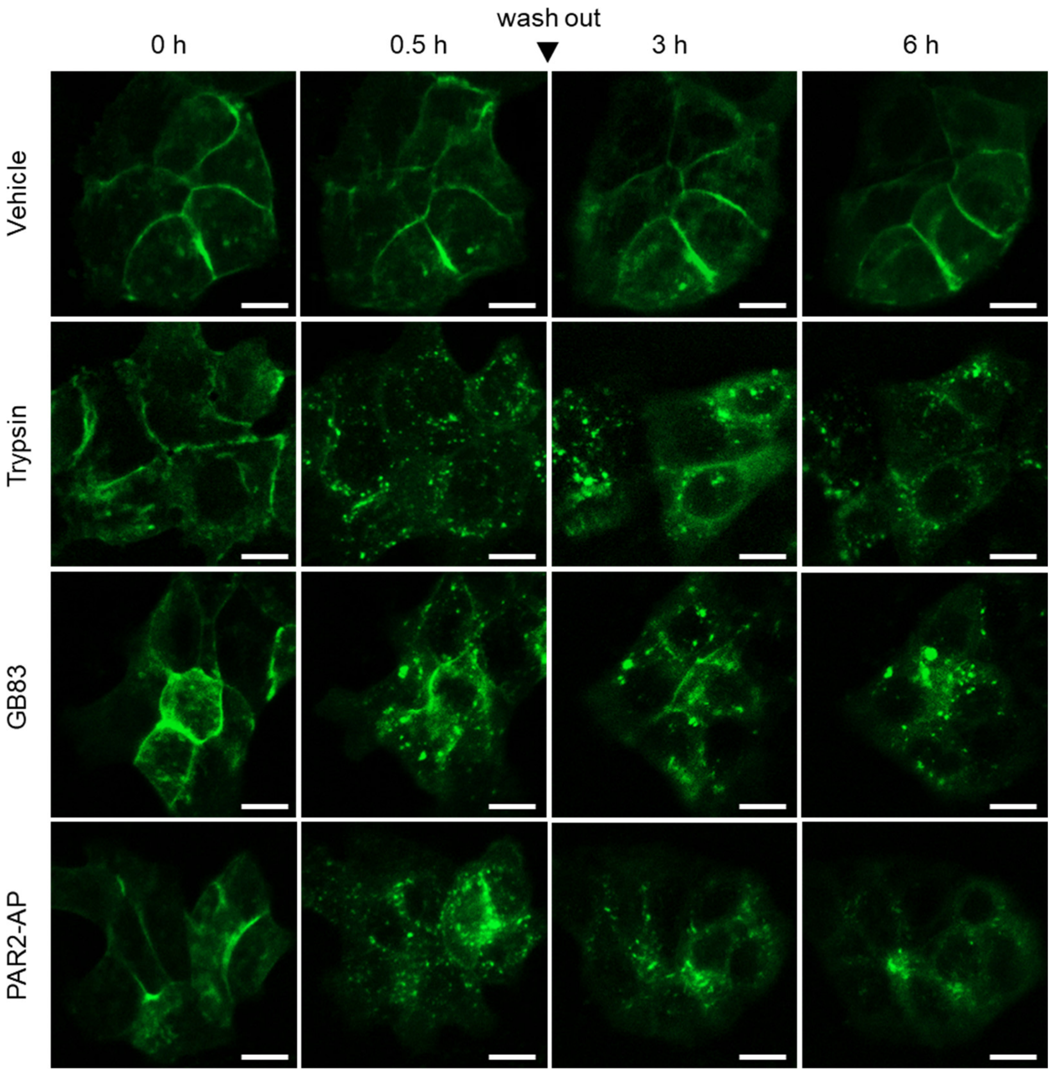

Figure S2. Effect of brefeldin A on the recovery of PAR2 from GB83, PAR2-AP and trypsin-induced endocytosis in HT29 cells expressing EGFP-tagged PAR2. Cells were pretreated with brefeldin A (200 nM) for 20 min, then treated with trypsin (30 U/mL), GB83 (30 μM) or PAR2-AP (30 μM) for 30 min, washed 3 times with PBS, and then the medium was replaced with brefeldin A (200 nM) containing medium. Cellular localization of EGFP-tagged PAR2 were observed at the indicated time points.
